# Hepatitis B Virus Prevalence and Mother-to-Child Transmission Risk in an HIV Early Intervention Cohort in KwaZulu-Natal, South Africa

**DOI:** 10.1093/ofid/ofad366

**Published:** 2023-07-15

**Authors:** Jane Millar, Gabriela Z L Cromhout, Noxolo Mchunu, Nomonde Bengu, Thumbi Ndung’u, Philip J Goulder, Philippa C Matthews, Anna L McNaughton

**Affiliations:** Department of Paediatrics, University of Oxford, Oxford, UK; HIV Pathogenesis Programme, The Doris Duke Medical Research Institute, University of KwaZulu Natal, Durban, South Africa; Department of Paediatrics and Child Health, University of KwaZulu Natal, Durban, South Africa; HIV Pathogenesis Programme, The Doris Duke Medical Research Institute, University of KwaZulu Natal, Durban, South Africa; HIV Pathogenesis Programme, The Doris Duke Medical Research Institute, University of KwaZulu Natal, Durban, South Africa; Department of Paediatrics, Queen Nandi Regional Hospital, Empangeni, South Africa; HIV Pathogenesis Programme, The Doris Duke Medical Research Institute, University of KwaZulu Natal, Durban, South Africa; Africa Health Research Institute, Durban, South Africa; Division of Infection and Immunity, University College London, London, UK; Department of Paediatrics, University of Oxford, Oxford, UK; Division of Infection and Immunity, University College London, London, UK; The Francis Crick Institute, London, UK; Department of Infectious Diseases, University College London Hospital, London, UK; Nuffield Department of Medicine, University of Oxford, Oxford, UK

**Keywords:** antiretroviral therapy, hepatitis B virus, HIV, PMTCT, South Africa

## Abstract

**Background:**

HIV and hepatitis B virus (HBV) prevalence are both high in KwaZulu-Natal, South Africa. HIV coinfection negatively affects HBV prognosis and can increase the likelihood of HBV mother-to-child transmission (MTCT). In an early HIV infant treatment intervention cohort of HIV-transmitting mother-child pairs in KwaZulu-Natal, we characterized maternal HBV prevalence and screened infants at risk.

**Methods:**

Infants were treated for HIV MTCT at birth, and combination regimens incidentally active against HBV were initiated within 21 days. Maternal samples (N = 175) were screened at birth for HBV infection (HBV surface antigen [HBsAg]), exposure to HBV (HBV anti-core IgG), and vaccination responses (HBV anti-S positive without other HBV markers). Infants of mothers who were HBV positive were screened for HBsAg at 1 and 12 months.

**Results:**

Evidence of HBV infection was present in 8.6% (n = 15) of maternal samples. Biomarkers for HBV exposure were present in 31.4% (n = 55). Evidence of HBV vaccination was uncommon in mothers (8.0%; n = 14). Despite prescription of antiretroviral therapy (ART) active against HBV, HBV DNA was detectable in 46.7% (7/15) of mothers who were HBsAg positive. Three mothers had HBV viral loads >5.3 log_10_ IU/mL, making them high risk for HBV MTCT. Screening of available infant samples at 1 month (n = 14) revealed no cases of HBV MTCT. At 12 months, we identified 1 HBV infection (1/13), and serologic evidence of vaccination was present in 53.8% (7/13) of infants.

**Discussion:**

This vulnerable cohort of HIV-transmitting mothers had a high prevalence of undiagnosed HBV. Early infant ART may have reduced the risk of MTCT in high-risk cases. Current HBV guidelines recommend ART prophylaxis, but these data underline the pressing need to increase availability of birth dose vaccines.

The World Health Organization Africa region bears a disproportionate burden of global hepatitis B virus (HBV), with an estimated 75 million individuals chronically infected in the region [1], of whom 2.6 million are living with HBV/HIV coinfection [2]. The KwaZulu-Natal (KZN) province in South Africa (SA) has a high HIV prevalence, reported as 40.9% in a 2019 national antenatal survey [3]. HBV prevalence in KZN was estimated to be 4.0% in a 2019 household survey, although prevalence was >2-fold higher among people living with HIV [4]. HIV coinfection can negatively affect HBV outcomes, with approximately 2.5-fold greater risk of HBV mother-to-child transmission (MTCT) [5, 6]. However, individuals with coinfection potentially benefit from shared treatment with nucleos/tide analogues active against both viruses, including lamivudine (3TC), tenofovir disoproxil fumarate (TDF), and emtricitabine [7, 8]. Antiretroviral therapy (ART) is recommended throughout pregnancies for women living with HIV, and this may also suppress HBV infection, mitigating MTCT risk [8].

High maternal HBV viral load (VL) and positive HBV e-antigen (HBeAg) status increase the risk of HBV MTCT [2]. World Health Organization guidelines advocate that nucleos/tide analogue treatment be started between weeks 24 and 28 of gestation when the maternal HBV VL is >5.3 log_10_ IU/mL [9]. Additional interventions are recommended, including HBV immunoglobulin in selected infants, alongside universal birth dose vaccine for neonates (given within the first 24 hours of life) [10]. HIV MTCT in SA has drastically reduced in recent years [11] and is most common in vulnerable individuals with the least interaction with health care. To investigate the impact of HIV coinfection and its treatment on HBV MTCT in KZN, we retrospectively screened maternal and infant serum samples from a cohort in which HIV MTCT was documented.

## METHODS

### Cohort and Management of HIV and HBV Infections

We retrospectively tested a cohort in which in utero HIV MTCT was documented from the Ucwaningo Lwabantwana (Learning From Children) study, established in KZN in 2015, to examine the impact of early initiation of combination ART (cART) on HIV infection in infants [12]. Local HIV/HBV management guidelines were adhered to [13, 14]. Mothers initiated lifelong ART at HIV diagnosis, but incomplete adherence to ART was typical [12]. All first-line treatments were cART, including at least 1 HBV active agent (typically TDF).

Infants were treated within 48 hours of life for HIV MTCT and switched to cART within 21 days. 3TC was included in all infant cART. Clinical data and blood samples from the mother-child pairs were collected at enrollment (within 21 days of delivery), monthly for 6 months, then every 6 months thereafter. Samples from the mothers were tested for HBV serology at birth, and infant 1- and 12-month samples were screened for HBV. Infants in this cohort should have received HBV immunization as part of a multivalent vaccine schedule starting at age 6 to 10 weeks but were unlikely to have received birth dose vaccination. HBV immunoglobulin is not routinely available in SA.

### HBV Serology Testing

Maternal HBV infection was determined by the presence of HBV surface antigen (HBsAg) and previous infection by the presence of total HBV anti-core IgG (anti-HBc) without HBsAg. Mothers were screened for anti-HBc IgM, which can be an indicator of recent infection. HBV vaccination was assumed if HBV anti-S (anti-HBs) was present in the absence of any other HBV biomarker (Supplementary Table 1). Samples testing HBsAg positive were further tested for HBeAg and HBV DNA quantification to determine risk of MTCT (Supplementary Figure 1 for testing approach). Infant samples were screened for HBsAg if the maternal sample was HBsAg positive. If at either time point they were HBsAg positive, HBV DNA was also tested. Anti-HBs was assessed in infants at 12 months of age who were at risk to determine vaccine-mediated immunity.

HBV testing was carried out by Neuberg Global Laboratories. Higher risk of MTCT was assumed in mothers who were HBeAg positive or had HBV DNA >5.3 log_10_ IU/mL [9]. Maternal age, ART, HIV VL, and CD4 count were recorded during the Ucwaningo Lwabantwana study [12]. Statistical analysis was performed via Stata version 16.1 (StataCorp) and Prism version 9.4 (GraphPad) with Kruskal-Wallis tests to determine statistical differences between groups.

### Ethics and Patient Consent Statement

The study was approved by the KZN Bioethics Research Ethics Committee and the Oxfordshire Research Ethics Committees (BF450/14). Written informed consent for the infant and mother's participation in the study was obtained from the mother or infant's legal guardian. Maternal study identifications (IDs) were anonymized for publication.

## RESULTS

### High Maternal HBsAg Prevalence Is Present in This Population

**Figure 1.**
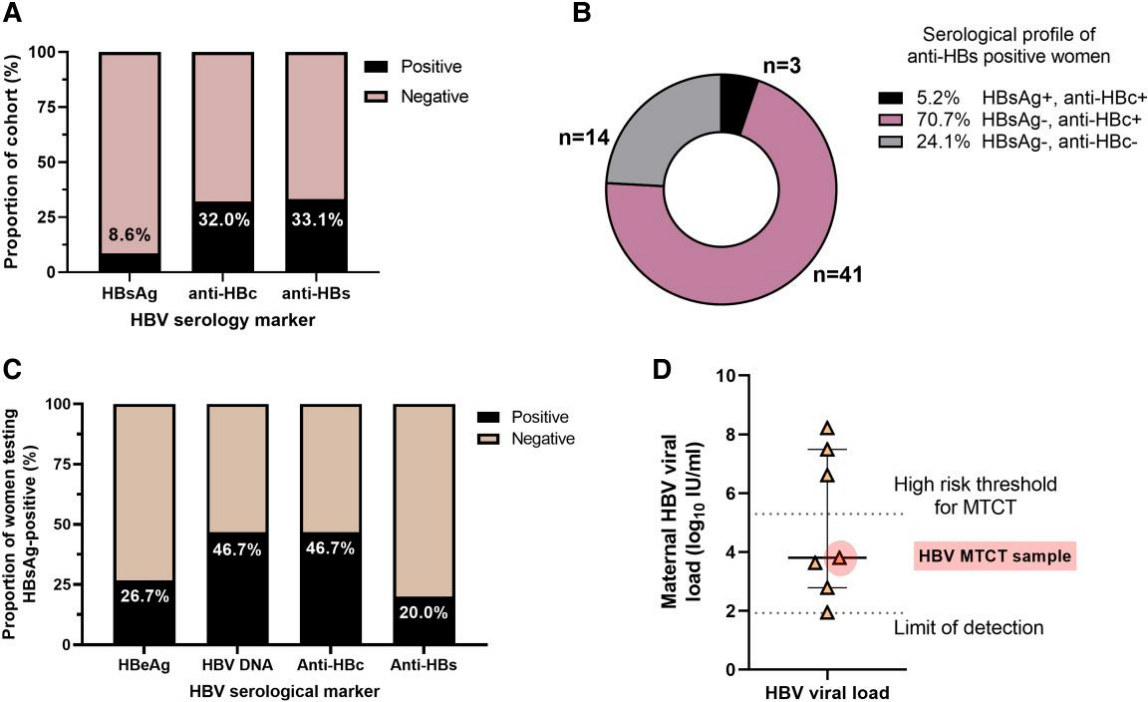
*A–D,* HBV serologic profiles of mothers in a cohort of HIV MTCT pairs in KwaZulu-Natal, South Africa. *A*, All women in the cohort (N = 175) were tested for HBV surface antigen (HBsAg), HBV anti-core IgG (anti-HBc), and HBV anti-S (anti-HBs). *B*, Serological profiles of anti-HBs positive women (n = 58). HBV vaccination was likely in women with HBsAg–/anti-HBc– status, with the presence of any other HBV biomarker (HBsAg and/or anti-HBc) suggesting that immunity is the result of previous infection. *C*, Additional testing of women who were HBsAg positive is presented (n = 15), which included HBV e-antigen (HBeAg) and HBV DNA. *D*, Seven women who were HBsAg positive had HBV viral loads above the limit of detection (1.92 log_10_ IU/mL). The viral load of the maternal sample from the HBV MTCT pair is highlighted in red. The high-risk threshold for MTCT of HBV is also indicated (5.3 log_10_ IU/mL). Data are presented as median (IQR). HBV, hepatitis B virus; MTCT, mother-to-child transmission.

This study included 175 mothers who were HIV positive, sampled between July 2015 and April 2021, of whom 15 (8.6%) were HBsAg positive (Figure 1*A*) at the time of delivery. Among these, 3 (20%) were not recorded as undergoing ART during pregnancy, with the other 12 receiving ART for varying amounts of time (Table 1). However, as evidenced by HIV MTCT in all cases, ART was inconsistent due to the diverse social vulnerabilities in these women, as previously described [15]. HBsAg-positive status was not associated with HIV VL or T-cell counts (Supplementary Figure 2), and women who were HBsAg positive were younger than women who were HBsAg negative (mean, 21.7 vs 25.5 years; Supplementary Table 2).

**Table 1.** Maternal HBV and HIV Virology and Serology Results Among Mother-Child Paired Samples Who Were HBsAg (n = 15) and Anti-HBc IgM (n = 1) Positive

	Maternal HBV Serology and Virology					Maternal HIV VL, CD4 and CD8 Counts, and Treatment at Delivery					Infant HBV Serology			Infant HIV Treatment		
Maternal Study ID	Anti-HBs	Anti-HBc	HBeAg	VL, Log_10_ IU/mL	HBV MTCT Risk	Proportion of Pregnancy on ART, %	HBV Active NA in Maternal Treatment^a^	Maternal HIV VL, Log_10_ Copies/mL	CD4, Cells/mL	CD8, Cells/mL	Infant HBsAg, 1 month	Infant HBsAg, 12 months	Infant Anti-HBs, 12 months	Infant PMTCT at Birth^b^	Infant cART^b^	Age at cART Initiation, days^c^
HBKN-109	+	+ [IgM+]	N/A	N/A	Unclear	69.5	TDF	3.3	653	1554	−	−	+	AZT	3TC/AZT/NVP	8–14
HBKN-26	−	+	−	1.94	Low	100.0	TDF	<1.30	479	1089	−	−	+	NVP/AZT	AZT/3TC/NVP	≤7
HBKN-61	−	−	−	<1.92	Low	45.9	TDF	<1.30	552	1358	−	−	−	AZT/NVP	AZT/3TC/NVP	8–14
HBKN-62	+	+	−	<1.92	Low	4.9	TDF	3.53	481	1717	−	−	+	NVP	AZT/3TC/NVP	≤7
HBKN-81	−	−	−	<1.92	Low	6.3	TDF	2.93	1183	946	−	−	+	NVP/AZT	3TC/AZT/NVP	8–14
HBKN-99	−	+	−	3.8	Low	100.0	3TC	5.64	331	727	−	+	+	NVP/AZT	3TC/AZT/NVP	15–21
HBKN-100	+	+	−	<1.92	Low	0.0	TDF	4.59	606	1349	−	−	+	NVP/AZT	3TC/AZT/NVP	≤7
HBKN-103	+	+	−	<1.92	Low	0.0	TDF	3.45	437	2213	−	−	−	NVP/AZT	3TC/AZT/NVP	≤7
HBKN-114	−	−	−	<1.92	Low	100.0	TDF	5.11	770	806	NS	NS	NS	AZT	3TC/AZT/NVP	≤7
HBKN-117	−	+	−	<1.92	Low	2.9	TDF	4.84	726	1154	−	−	+	AZT/NVP	3TC/ABC/KAL	≥22
HBKN-149	−	−	−	3.63	Low	12.4	TDF	4.95	333	1273	−	−	+	NVP	3TC/NVP/AZT	8–14
HBKN-160	−	−	−	<1.92	Low	0.0	TDF	<1.30	389	967	−	−	−	AZT/NVP	3TC/AZT/NVP	8–14
HBKN-72	−	−	+	2.78	Low	57.5	3TC	4.08	50	442	−	NS	NS	NVP/AZT	3TC/AZT/NVP	≤7
HBKN-05	−	−	+	>8.22	High	100.0	TDF	5.40	252	777	−	NS	NS	NVP/AZT	3TC/NVP/AZT	≤7
HBKN-118	−	+	+	6.62	High	53.8	TDF	<1.30	292	722	−	−	−	AZT/NVP	3TC/AZT/NVP	15–21
HBKN-159	−	−	+	7.49	High	100.0	TDF	4.60	109	375	−	−	−	AZT/NVP	3TC/AZT/NVP	≤7

HBV MTCT risk was based on maternal HBV viral load, with the World Health Organization threshold >5.3 log10 IU/mL used to identify high risk pregnancies [9]. One sample (HBKN-109) tested HBsAg negative and anti-HBc IgM positive and interpretation was unclear, so samples from the paired infant were screened. One mother-child pair had no infant samples available for testing at either time point; another sample had insufficient volume at 12 months; and another was lost to follow-up.

Abbreviations: anti-HBc, HBV anti-core IgG; anti-HBs, HBV anti-S; ART, antiretroviral therapy; cART, combination ART; HBeAg, HBV e-antigen; HBV, hepatitis B virus; HBsAg, HBV surface antigen; MTCT, mother-to-child transmission; N/A, not applicable; NA, nucleos/tide analogue; NS, no sample available; PMTCT, prevention of MTCT; VL, viral load.

a Maternal treatments included TDF (tenofovir) or 3TC (lamivudine).

b Infant HIV PMTCT included AZT (zidovudine) and/or NVP (nevirapine). cART also included 3TC, ritonavir-boosted lopinavir (referred to as Kaletra [KAL]), and abacavir (ABC).

c The study aimed to prescribe infant cART within 21 days of life.

### High Risk of HBV MTCT Is Present Despite Prescription of HBV-Active ART

Among the 15 mothers testing HBsAg positive, 4 (26.7%) were HBeAg positive and 7 (46.7%) were HBV DNA positive at delivery (Table 1, Figure 1*C*). All women who were HBeAg positive had detectable HBV DNA; of these, 3 of 4 women had VL classified as high risk for MTCT (Figure 1*D*). CD4+ T-cell counts were lower in women testing HBV DNA positive than in their counterparts who were virologically suppressed (263.7 vs 643.0 cells/mm^3^; Supplementary Table 2). One mother (ID HBKN-118) had an HBV VL of 6.6 log_10_ IU/mL and was aviremic for HIV (Table 1), indicating a similar clinical phenotype to previously reported cases of HBV drug resistance in SA where suppression of HIV is a proxy for adequate adherence to therapy [16].

### Other Maternal HBV Biomarkers

Approximately a third of mothers in the study were anti-HBc positive (32.0%, 56/175), indicating serologic evidence of exposure to HBV. However, among the HBsAg-positive samples, only 7 of 15 (46.7%) were anti-HBc IgG positive. One mother was anti-HBc IgM, anti-HBs, and anti-HBc IgG positive, potentially suggesting recent exposure and clearance or reactivation.

Anti-HBs was detected in 33.1% mothers (58/175) (Figure 1). Anti-HBs was rare among the women who were HBsAg positive, with just 3 (20%) of these women testing anti-HBs positive. These women were all anti-HBc positive and HBeAg negative, and HBV DNA was below the limit of detection. Only 14 of 58 women had an anti-HBs–only profile, reflecting a low prevalence of vaccine-mediated immunity, at just 8.0% in the cohort overall (Figure 1*B*). The presence of anti-HBs and anti-HBc was not associated with HIV VL or T-cell counts (Supplementary Figures 3, 4).

### HBV Detected in 1 Infant at Age 12 Months in a Mother With Detectable HBV and HIV Viraemia at Birth

All screened infants were HBV negative at 1 month (n = 14; no sample for 1 infant), and 1 of 13 (7.7%) infants was HBV positive at 12 months (maternal ID HBKN-99) with an HBV VL of 3.1 log_10_ IU/mL. Samples were unavailable for 3 infants at 12 months of age. At birth, the mother of the infant who was HBV positive was HBeAg negative, with an HBV VL of 3.8 log_10_ IU/mL, and was recorded as taking TDF-based therapy throughout pregnancy but had a detectable HIV VL of 5.6 log_10_ copies/mL. Of note, this infant did not receive 3TC containing cART until 21 days of life, as compared with the overall median of 6.5 days (IQR, 2.3–12.8) among other infants. Despite the ongoing prescription of cART, at 12 months the infant had an HIV VL >8.0 log_10_ HIV RNA copies/mL and did not achieve HIV plasma viral suppression until 18 months of age, suggesting poor adherence. HBV infection was not detected in any of the infants of the mothers deemed high transmission risk, although 1 mother-child pair (maternal ID HBKN-05) was lost of follow-up after initial enrollment. The infant from the mother who was anti-HBc IgM positive was also screened at 1 and 12 months, and both these infant samples tested negative (Table 1).

### Infants With Vertical HIV Infection Have a Low Rate of Vaccine-Mediated Immunity to HBV Infection

All available 12-month infant samples that were screened for HBsAg were screened for anti-HBs (n = 13; including the infant of the mother who was IgM positive) to look for evidence of HBV vaccination (Table 1). An anti-HBs–only serologic profile was observed in 7 of 13 (53.8%) infants. The infant who was HBsAg positive also tested anti-HBs positive (1/13), and the remaining samples were anti-HBs negative (5/13). This suggests that 7 of 13 infants in this cohort received infant vaccination, although we cannot exclude the possibility that partial vaccination series were delivered or that, despite vaccination, anti-HBs titers were not mounted due to HIV coinfection.

## DISCUSSION

A high HBV prevalence (8.6%) was observed in this vulnerable cohort of mothers. Among the coinfected group, 20% were classified as high risk for HBV MTCT based on virologic parameters, reaching this threshold despite the availability of ART active against HBV. Despite these risks, all infants in the cohort from mothers infected with HBV tested HBV negative at birth.

A single HBV infection was identified in an infant at 12 months of age, born to a mother who was not classified as high risk [9]. However, women with HIV/HBV coinfection should be prescribed ART regardless of HBV VL [17, 18], and poorly controlled HIV infection is itself a risk factor for HBV transmission in this case. It is noteworthy that the majority of data informing these guidelines stem from Asia [19] and that studies in Africa have documented HBV MTCT with a lower HBV VL and in women who are HBeAg negative [20, 21]. It is possible this early infant intervention for neonates with HIV may have minimized HBV MTCT events in this cohort.

Serology suggested a lack of vaccine-mediated immunity in 5 of 13 infants tested, indicating that a high proportion of infants remain at risk of horizontal transmission later in childhood. Previous work has shown that vaccinated infants who are HIV positive typically produce protective anti-HBs titers until approximately 1 year of age but that titers wane rapidly in the following years [22], suggesting that infants without responses in our study had missed or incomplete vaccine courses. Our data underscore the importance of (1) combining antenatal HBV screening with maternal prophylaxis and (2) starting infant vaccination with a timely birth dose vaccine and follow-up doses during infancy.

Serologic evidence of maternal HBV vaccination was low at just 8.0%. However, it is possible that a higher proportion received childhood vaccination but that vaccine-mediated antibody titers had waned over time in the context of HIV infection [22]. Anti-HBc IgG is assumed to persist for life, making it useful for population-based screening [23]. Over half the women who were HBsAg positive in this cohort were anti-HBc negative, with recent infection unlikely, suggesting that HIV infection is compromising the generation of anti-HBc antibodies in these women. This serologic profile has been described in other coinfected cohorts [24, 25] associated with HIV (especially with low CD4 counts) and in patients undergoing solid organ transplants, suggesting an association with immune compromise [26]. In populations where HIV is coendemic, screening based on anti-HBc may therefore underestimate the proportion of the population exposed.

Women who tested HBsAg positive were on average 4 years younger than other women in the cohort, suggesting that younger women were particularly vulnerable to HBV MTCT. Previous research has indicated that adolescent and younger mothers are generally less engaged with the HIV continuum of care for the prevention of MTCT than older women, placing their infants at greater risk of HIV and HBV infection [27, 28].

### Limitations

The number of women in the study who were HBV positive was small, with a single mother-child case of HBV MTCT identified, making findings difficult to generalize. HBV sequencing to confirm linkage in the mother-child pair would have been helpful, but this was not feasible: both had relatively low HBV DNA (∼3.0 log_10_ IU/mL), which makes sequencing challenging [29]. Sequencing would also have been informative to better understand the clinical phenotype in the mother who had undetectable HIV but a high HBV VL.

Occult HBV, where individuals are HBsAg negative but HBV DNA positive [30], has been reported to be more common among individuals who are HIV positive [31]. The testing algorithm used in our study did not screen for occult HBV, and it is therefore possible that numerous HBV cases were overlooked. Further work on the prevalence of occult HBV and the potential risk of HBV MTCT in these cases would be informative, but HBV DNA testing remains costly relative to serologic testing, limiting widespread use in resource-poor settings [32].

## CONCLUSIONS

Our study indicates a high HBV prevalence among mothers living with HIV in KZN, putting their pregnancies at risk of HBV MTCT. It is important to avoid complacency in assuming that prescription of ART active against HIV and HBV prevents against transmission events. However, maternal ART and the early ART treatment of infants likely contributed to reducing the chances of infant HBV infections in mothers at high risk. A role for the increased use of maternal prophylaxis and potential neonatal postexposure prophylaxis in infants at high risk should be considered in addition to robust vaccination (birth dose and follow-up in infancy). Interdisciplinary interventions are required to support disadvantaged women who are at high risk of MTCT to enhance screening, treatment, and prophylaxis. These interventions are crucial to support progress toward international elimination goals for HBV.

## Supplementary Material

ofad366_Supplementary_Data

## Supplementary Data

Supplementary materials are available at *Open Forum Infectious Diseases* online. Consisting of data provided by the authors to benefit the reader, the posted materials are not copyedited and are the sole responsibility of the authors, so questions or comments should be addressed to the corresponding author.

## Notes

***Author contributions.*** All authors contributing to the study were given the opportunity to comment on the manuscript and approved the final submitted version of the manuscript.

***Acknowledgments.*** We would like to acknowledge all the women and their infants who have participated in the Ucwaningo Lwabantwana (Learning From Children) study over the years and the many health care workers who have supported the families throughout.

***Data availability.*** HBV serology data for all 175 mothers and infants tested as a part of the study are available on Figshare: https://doi.org/10.6084/m9.figshare.22259224.

***Financial support.*** This study was funded by an Oxford University John Fell Fund award (0008410) held by A. L. M. Supplementary testing was funded by the Wellcome Trust. P. C. M. is funded by Wellcome (grant 110110/Z/15/Z), the Francis Crick Institute, and UCLH Biomedical Research Centre.
