# Supplementary material for: Hepatitis B Virus Prevalence and Mother-to-Child Transmission Risk in an HIV Early Intervention Cohort in KwaZulu-Natal, South Africa
Source: Open Forum Infect Dis. 2023 Jul 15;10(8):ofad366. doi: 10.1093/ofid/ofad366 (PMC10400149; doi:10.1093/ofid/ofad366)
Supplement: ofad366_Supplementary_Data [file ofad366_supplementary_data.docx]

**Hepatitis B Virus (HBV) prevalence and mother-to-child transmission risk in an HIV early intervention cohort in KwaZulu-Natal, South Africa**

**Supplementary data file**

Jane Millar, Gabriela Z.L. Cromhout, Noxolo Mchunu, Nomonde Bengu, Thumbi Ndung’u, Philip J Goulder, Philippa C Matthews, Anna L McNaughton

**Supplementary Tables**

**Supplementary Table 1; Combinations of HBV serology result and the clinical interpretation.** Individuals in the study were also tested for anti-HBc IgM, which is typically considered a marker for acute infection although it is also observed in reactivated infections.

| **HBV serology result** | | | **Interpretation** |
| --- | --- | --- | --- |
| **HBsAg** | **Anti-HBs** | **Anti-HBc (IgG)** |  |
| **+** | **+/-** | **+/-** | **HBV infection** |
| **-** | **+/-** | **+** | **Previous infection** |
| **-** | **+** | **-** | **HBV negative, vaccinated** |
| **-** | **-** | **-** | **HBV negative, not vaccinated** |

**Supplementary Table 2; Age and HIV associated factors and association with HBsAg and presence of HBV DNA.** The age of the mothers in the cohort, proportion of their pregnancy on antiretroviral therapy (ART), HIV viral loads (VL) and CD4 counts are given for HBsAg positive and negative women, and HBV DNA positive and negative women.

| **Among KZN Ucwaningo Lwabantwana mothers (n=175)** | | | |  |  |
| --- | --- | --- | --- | --- | --- |
|  | **HBsAg positive**  **(n=15)** | | **HBsAg negative**  **(n=160)** | |  |
|  | **Mean** | **95%CI** | **Mean** | **95%CI** | **p** |
| **Age (yrs)** | 21.7 | 20.3-23.2 | 25.5 | 24.7-26.4 | 0.009 |
| **Pregnancy on ART (%)** | 45.6 | 23.0-68.1 | 43.4 | 37.4-49.5 | 0.927 |
| **HIV VL (log_10_ copies/ml)** | 4.9 | 4.0-5.1 | 4.9 | 4.6-5.1 | 0.733 |
| **CD4 count (cells/mm^3^)** | 466 | 321.8-610.2 | 518.8 | 473.6-564.0 | 0.481 |
| **Among HBsAg positive mothers only (n=15)** | | |  |  |  |
|  | **HBV DNA positive**  **(n=7)** | | **HBV DNA negative (n=8)** | |  |
|  | **Mean** | **95%CI** | **Mean** | **95%CI** | **p** |
| **Age (yrs)** | 21.5 | 18.8-24.3 | 22.0 | 19.8-24.2 | 0.745 |
| **Pregnancy on ART (%)** | 74.8 | 42.8-100.0 | 20.0 | 0.0-49.9 | 0.011 |
| **HIV VL (log_10_ copies/ml)** | 4.0 | 2.2-5.6 | 3.4 | 2.1-4.6 | 0.354 |
| **CD4 count (cells/mm^3^)** | 263.7 | 129.6-397.9 | 643.0 | 429.3-856.7 | 0.003 |

**Supplementary Figures**

**Supplementary figure 1; Testing approach used to screen mothers and infants in the HBV study, indicating when further testing was required.** Maternal samples were for HBsAg, anti-HBs, anti-HBc IgM and total anti-HBc. Mothers were considered HBV-positive if HBsAg was detected and these samples were further tested for HBV DNA and HBeAg. Infants of HBV-positive mothers, or those testing HBV IgM positive, were considered ‘at-risk’ and only these samples were tested. Infant samples were only tested for anti-HBs at 12 months of age, as infant vaccination typically occurs after 6 weeks of age.

**Supplementary Figure 2; (A) HIV viral load, (B) CD4 count and (C) CD8 count of mothers stratified by HBsAg status.** Median and interquartile ranges are indicated, and there was no significant difference between HBsAg positive and negative women for any biomarkers.

**Supplementary Figure 3; (A) CD4 counts and (B) HIV viral loads stratified by anti-HBs status.** Median and interquartile ranges are indicated, and there was no significant difference between anti-HBs positive and negative women for either CD4 count or HIV viral loads.

**Supplementary Figure 4; (A) HIV viral load, (B) CD4 counts and (C) CD8 counts stratified by anti-HBc status.** All anti-HBc positive women were compared with HBsAg-positive/anti-HBc negative women. Median and interquartile ranges are indicated, and there was no significant differences observed for any of the biomarkers.
